# Targeting QRICH1 suppresses epithelial-mesenchymal transition and tumor growth in liver cancer through the Connexin43/USP1 mediated Snail1 stabilization

**DOI:** 10.7150/ijbs.133080

**Published:** 2026-06-10

**Authors:** Su-Yeon Park, Bum-Sang Shim, Bonglee Kim, Sung-Hoon Kim

**Affiliations:** Cancer Molecular Targeted Herbal Research Laboratory, College of Korean Medicine, Kyung Hee University, 1 Hoegi-dong, Dongdaemun-gu, Seoul 02447, South Korea.

**Keywords:** QRICH1, Connexin43, USP1, Snail1, gap junction intercellular communication, epithelial-mesenchymal transition

## Abstract

Although glutamine-rich protein 1 (QRICH1) has been implicated in endoplasmic reticulum stress-associated epithelial-mesenchymal transition (EMT), its mechanistic role in liver cancer progression remains unclear. QRICH1 expression was analyzed in public datasets and clinical liver cancer specimens. Gain- and loss-of-function approaches were performed to assess proliferation, migration, invasion, and EMT signaling *in vitro*, and tumorigenicity was evaluated using a xenograft mouse model. QRICH1 was significantly overexpressed in liver cancer tissues with poor clinical outcomes. QRICH1 silencing markedly suppressed cell proliferation, migration, invasion, and EMT, accompanied by decreased Snail1 and ZEB1 expression and restoration of Connexin43 (GJB1). Co-immunoprecipitation and immunofluorescence analyses demonstrated that QRICH1 physically interacted and colocalized with Snail1 and USP1. Molecular docking further supported stable binding interfaces among these proteins. Ubiquitination and cycloheximide chase assays revealed that QRICH1 enhanced Snail1 protein stability through USP1-mediated deubiquitination. Functionally, QRICH1 suppressed gap junction intercellular communication, consistent with reduced Connexin43 expression, but not Connexin26 and Connexin32. *In vivo*, QRICH1 knockdown significantly inhibited tumor growth and reduced the expression of USP1, Snail1, PCNA, VEGF, and N-cadherin, while increasing E-cadherin and Connexin43. Collectively, these findings identify QRICH1 as a key oncogenic regulator that promotes liver cancer progression by stabilizing Snail1 via USP1-dependent deubiquitination and disrupting Connexin43-mediated gap junction signaling.

## Introduction

Liver cancer, predominantly hepatocellular carcinoma (HCC), remains a leading cause of cancer-related mortality worldwide, driven by frequent recurrence, early metastasis, and limited therapeutic durability 1, 2. Although advances in surgical resection as well as targeted and immune-based therapies have improved outcomes in selected patients, the prognosis of advanced HCC continues to be unsatisfactory, highlighting a persistent unmet need to identify molecular programs that govern tumor progression 3. Among the biological processes associated with aggressive HCC behavior, epithelial-mesenchymal transition (EMT) has emerged as a highly dynamic cellular program that promotes tumor cell invasion, dissemination, stem-like properties, and resistance to chemotherapy 4. EMT is regulated by interconnected transcriptional networks involving factors such as Snail1, ZEB1, and Twist, which repress epithelial markers including E-cadherin while activating mesenchymal gene expression, such as N-cadherin and vimentin 5.

Among these regulators, Snail1 is considered one of the most potent EMT-inducing transcription factors and is subject to stringent control by intracellular signaling cascades and the ubiquitin-proteasome system (UPS) 6. Deubiquitinating enzymes (DUBs), particularly ubiquitin-specific protease 1 (USP1), have been reported to stabilize Snail1 by counteracting its proteasomal degradation, thereby sustaining EMT programs and malignant progression 7, 8. In parallel, among 21 Connexin family members 9, Connexin43 (Cx43), a principal component of gap junction intercellular communication (GJIC), exerts multifaceted and context-dependent roles in tumor biology 10. While intact Cx43-mediated GJIC is generally associated with tumor-suppressive functions 11, aberrant expression or dysfunction of Cx43 has been linked to disrupted cell-cell communication, EMT activation, and metastatic behavior in multiple cancer types, including HCC 8, 12.

Glutamine-rich protein 1 (QRICH1) has been identified as a transcriptional regulator associated with the caspase recruitment domain (CARD) 13, and is known to participate in endoplasmic reticulum (ER) stress responses 14. QRICH1 has also been implicated in cellular survival pathways, including ATF6 signaling, 15, as well as in immune-related processes such as T-cell apoptosis 16, 17. Recent studies suggest that QRICH1 may influence EMT-related programs; however, its biological functions and mechanistic roles in liver cancer remain largely undefined. In particular, whether QRICH1 contributes to EMT regulation through modulation of Snail1 stability, USP1 activity, Connexin43 expression, or GJIC has not yet been explored.

Despite the well-established importance of EMT in driving metastasis 5, 18, the upstream regulatory networks controlling EMT-inducing transcription factors in liver cancer are still incompletely understood. Snail1 functions as a master regulator of EMT, and its protein stability is tightly governed by ubiquitin-dependent proteolysis 19. Nevertheless, the molecular determinants that coordinate Snail1 turnover and its interaction with deubiquitinases such as USP1 in HCC remain poorly characterized.

Accumulating evidence reveals that connexins can function as tumor suppressors by maintaining gap junctional intercellular communication and inhibiting proliferation, while in certain contexts they may also facilitate tumor progression depending on cellular context and microenvironmental cues 20, 21. Although Connexin43 is frequently downregulated in colorectal and breast cancers 11, 22, the mechanistic links between EMT transcriptional regulators and GJIC dysregulation in liver cancer have yet to be elucidated.

Therefore, this study aimed to characterize the expression pattern of QRICH1 in liver cancer tissues and cell lines, elucidate its functional and mechanistic roles in EMT regulation, and determine whether QRICH1 modulates the Connexin43-Snail1 and USP1-Snail1 signaling axes in both *in vitro* liver cancer models and *in vivo* xenograft system.

## Materials and Methods

### Cell lines and cell culture

The liver cancer cell lines Huh7, SK-Hep-1, HepG2, SNU-449 and human embryonic kidney cell line HEK293 were obtained from the Korean Cell Line Bank (KCLB, Seoul, Korea). Huh7 and SNU-449 cells were cultured in RPMI 1640 medium (Welgene, Gyeongsan, Korea) with 10 % fetal bovine serum (FBS) and 1 % antibiotic solution (100 units/ml penicillin and 100 µg/ml streptomycin). SK-Hep-1 cells were cultured in Dulbecco's Modified Eagle's Medium (DMEM) (Welgene, Gyeongsan, Korea) with 10 % fetal bovine serum (FBS) and 1 % antibiotic solution (100 units/ml penicillin and 100 µg/ml streptomycin). HepG2 and HEK293 cells were cultured in Minimum Essential Medium (MEM) (Welgene, Gyeongsan, Korea) with 10 % fetal bovine serum (FBS) and 1 % antibiotic solution (100 units/ml penicillin and 100 µg/ml streptomycin). All cells were maintained at 37 °C in an atmosphere of 5% CO_2_ and were determined to be mycoplasma-free free of mycoplasma contamination.

### TCGA data analysis and Kaplan-Meier survival rates

QRICH1 mRNA expression was analyzed by using human liver cancer clinical data from the Cancer Genome Atlas (TCGA) by using R studio program (MA, USA). Also, the rates of disease-free survival (DFS) and overall survival (OS) were determined from survival plot databases using the Kaplan-Meier method 23.

### cDNA microarray measurement and data analysis

As previously described 24, Total RNAs were isolated from Huh7 cells transfected with the siRNA -Control or siRNA-QRICH1 for 48 h by using Trizol and RNA quality was determined by RNA 6000 Nano Chip (Agilent Technologies, NH, Netherlands). For control and test RNAs, the construction of library was performed using QuantaSeq 3' mRNA-Seq Library Prep kit (Lexogen, Vienna, Austria) according to the manufacturer's protocol. In brief, 500 ng total RNAs were subjected to reverse transcription and then the mRNA expression profiling and gene classification were analyzed based on DAVID program and Medline databases. All experiments were conducted by e-Biogen Corporation (Seoul, Korea). The mRNA-sequencing data used in this study (GSE315229) are available from the NCBI public repository (https://www.ncbi.nlm.nih.gov/geo/query/acc.cgi?acc=GSE315229).

### Tissue microarray and immunohistochemistry

The human liver tumor tissue microarray (LV243) purchased from Biomax (MD, USA). As previously described 25, mouse tumors were fixed with 4% paraformaldehyde, dehydrated, embedded in paraffin and sectioned by 5 μm. Subsequently, the tumor sections were deparaffinized, rehydrated, and subjected to antigen retrieval in citrate buffer (pH 6.0). The sections were blocked with 1% BSA and 3% H₂O₂ in PBS for 1 h at room temperature, followed by overnight incubation with anti-QRICH1 (1:200) (# PA5-58052, Thermo Fisher Scientific, MA, USA), USP1 (1:300, 14346-1-AP, Proteintech, IL, USA), Snail1 (1:1000, sc-271977, Santa Cruz, TX, USA), PCNA (1:1000, sc-56, Santa Cruz, TX, USA), VEGF (1:500, sc-152, Santa Cruz, TX, USA), E-Cadherin (1:400, 3195s, Cell Signaling Technology, MA, USA) and N-Cadherin (1:200, #13116s, CST, MA, USA) at 4 °C. After washing with PBS, the sections were incubated with secondary antibody at room temperature for 1 h and developed using 3, 3-diaminobenzidine (DAB) solution. Finally, the images of slides were captured using a Leica SCN400 (Leica, Hesse, Germany). To evaluate staining intensity, the intensity score was categorized into four grades: 0, no staining; 1, weak staining; 2, moderate staining; and 3, strong staining. The H-score was calculated for each sample using ImageJ software with the following formula: H-score = (1 × % of weakly stained cells) + (2 × % of moderately stained cells) + (3 × % of strongly stained cells).

### Small interfering RNA (siRNA) and plasmid transfection

To induce transient knockdown, Huh7, SK-Hep-1 cells were seeded in 6-well plates and cultured overnight to reach 60% confluence, then transfected with untreated control siRNA or QRICH1 siRNA or USP1 siRNA using INTERFERin® (Polyplus, Bas-Rhin, France) for 48 h. All siRNA obtained from Bioneer (Daejeon, Korea) and siRNA transfection was performed according to the manufacturer's protocol. Expression plasmids for QRICH1 (Cat. No. HG26269-CF) and USP1 (Cat. No. HG20115-CM) were purchased from Sino Biological (China). Snail1 (Cat. No. #16218), HA-Ubiquitin (Cat. No. #18712), various ubiquitin mutants (WT, K6, K11, K27, K29, K33, K48 and K63) were acquired from Addgene (MA, USA). Cells for expression were transfected with plasmid using TurboFect™ (Thermo Fisher Scientific, MA, USA) for 48 h according to manufacturer's instructions.

### Cell viability assay

To perform the cell viability assay, cells were transfected with the specific siRNA and then seeded in 96-well plates at a density of 2 × 104 cells per well in 200 µl of medium. After 24 h, 48 h and 72 h, the plates were treated with 20 μl of 1 mg/ml 3-(4,5-dimethylthiazol-2-yl)-2,5-diphenyltetrazolium bromide (MTT) solution (Merck KGaA, Germany) and incubated at 37 ℃ for 3 h. The supernatant was carefully aspirated, and 100 μl of DMSO (Ducksan, Korea) was added to each well. The optical density was measured at 570 nm using a microplate spectrophotometer (Bio-rad, CA, USA).

### Colony formation assay

As previously described 26, Huh7 and SK-Hep-1 cells treated with siRNA were seeded in 6-well plates at a density of 1000 cells per well in 2 ml medium containing 10 % FBS. The culture medium was changed every three days. After two weeks of culture at 37 ℃ with 5 % CO, the cells were fixed and stained with Diff-Quik solution kit (Sysmex, Kobe, Japan). The wells were washed three times with PBS, dried, and images were captured. The number of individual colonies was then counted. For overexpression studies, SNU-449 and HEK293 cells were transfected with QRICH1 vector for colony formation assay.

### Wound healing assay

As previously described 27, Huh7, SK-Hep-1, SNU-449 and Hek293 cells transfected with siRNA or overexpression vector were seeded in 6-well plates and allowed to reach approximately 100% confluence. A 200 µl pipette tip was used to create a scratch in the monolayer. Images of the scratched area were captured at the same location at 0, 24, and 48 h using a microscope. And then the migratory activity of the cells was assessed.

### Immunofluorescence

As previously described 28, Huh7 and SK-Hep-1 cells grown to 4-well plates were fixed using a 4% formaldehyde solution, followed by permeabilization using 0.25% Triton X-100. Subsequently, the cells were blocked with 3% BSA in PBS for 1 h at room temperature. The cells were then probed with the primary antibodies of QRICH1 (1 μg; #PA5-58052, Thermo Fisher Scientific, MA, USA), Snail1 (1:500; sc-271977, Santa Cruz, TX, USA) and Connexin43 (1:50; #3512, CST, MA, USA) at 4℃ overnight. And then the cells were exposure to secondary antibodies of Alexa 488 Goat-anti-rabbit (1:1000; A11008, Thermo Fisher Scientific, MA, USA) and Alexa 546 Goat-anti-mouse (1:500; A11003, Thermo Fisher Scientific, MA, USA) for 2 h at room temperature. F-actin staining was performed using a dye from Thermo Fisher Scientific (Cat. A12379) following the manufacturer's instructions. The cells were stained with DAPI (10 μg; Sigma-Aldrich, MO, USA), mounted in mounting medium (Vector Laboratories, CA, USA) and captured images using FLUOVIEW FV10i confocal microscope (Olympus, Tokyo, Japan).

### Western blotting

As previously described 29, whole-cell lysates were isolated using RIPA lysis and extraction buffer (Thermo Fisher Scientific, MA, USA) supplemented with protease inhibitor (Sigma-Aldrich, MO, USA) and phosphatase inhibitor cocktail (Sigma-Aldrich, St. Louis, MO, USA). Subsequently, proteins from the lysates were quantified using a DC protein assay kit (Bio-Rad, CA, USA). Protein samples were boiled, and equal amounts of protein (15 μg per lane) were loaded onto an SDS-PAGE gel and transferred onto a nitrocellulose membrane (GE healthcare, MA, USA). The membranes were blocked with Smart-Block™ 5 min-Fast Blocking Buffer (BWB-0500, Biomax, Guri, Korea) for 5 min at room temperature, followed by overnight incubation at 4°C with the following primary antibodies: QRICH1 (ab241574; Abcam, Cambridge, UK), USP1 (#8033; CST, MA, USA), Snail1 (#3879; CST, MA, USA), E-cadherin (#3195; CST, MA, USA0, N-cadherin (#13116; CST, MA, USA), VEGF (SC-152; Santa Cruz, TX, USA), ZEB1 (#3396; CST, MA, USA), Ubiquitin (#20326; CST, MA, USA), ZO-1 (#8193; CST, MA, USA), Connexin43 (#3512; CST, MA, USA), Connexin32 (SC-59948; Santa Cruz, TX, USA), Connexin26 (SC-293223; Santa Cruz, TX, USA), cGAS (#15102; CST, MA, USA), Flag-tag (sc-166355; Santa Cruz, TX, USA), Myc-tag (#2276; CST, MA, USA), HA-tag (sc-7392; Santa Cruz, TX, USA) and β-actin (A1978; Sigma-Aldrich, MO, USA). And then the membranes were washed three times with TBST for 10 min, and incubated with horseradish peroxidase (HRP)-conjugated secondary goat anti-mouse IgGκ (sc-516102; Santa Cruz, TX, USA) or goat anti-rabbit IgG (#7074; CST, MA, USA) antibodies. The membranes were exposed to enhanced chemiluminescence (ECL) Western blotting detection reagent and were detected using the Amersham Imager 600 (Cytiva, BW, Germany).

### RNA isolation and Quantitative Real-time PCR (qRT-PCR)

As previously described 30, total RNA was extracted from cells transfected with various siRNA or QRICH1 plasmid using QIAzol (Qiagen, MD, USA). 2 ug of the total RNA samples were synthesized to complementary cDNA with oligo dT (Bioneer, Daejeon, Korea), dNTP (Takara, Kyoto, Japan) and M-MLV-Reverse transcriptase (Enzynomics, Daejeon, Korea). Quantitative real-time PCR (qRT-PCR) were performed using a Light cycler TM (Roche, Basel, Swiss) according to manufacturer's protocol. All primers were synthesized by Bioneer (Daejeon, Korea) and their sequences were as follows:

QRICH1 F: 5'-CACCAGTGTTCAGCCACAAACC-3',

QRICH1 R: 5'-GGTTGCTCAGTAGGCTGGTGAA-3',

SNAI1 F: 5'-TGCCCTCAAGATGCACATCCGA-3',

SNAI1 R: 5'-GGGACAGGAGAAGGGCTTCTC-3',

USP1 F: 5'-GCTCTAAAGGATGAAGCCAATCAA-3',

USP1 R: 5'-ACTAGCCTGGAGCTGTTCAACC-3',

GAPDH F: 5'-GTCTCCTCTGACTTCAACAGCG-3',

GAPDH R: 5'-ACCACCCTGTTGCTGTAGCCAA- 3'

The expression levels of QRICH1, SNAI1 and USP1 were determined using the 2-ΔΔCt method and normalized to the housekeeping gene GAPDH.

### Scrape loading/dye transfer assay

As previously described 31, to measure gap junction activity, Huh7 and SK-Hep-1 cells were grown in a four-well plate to approximately 100% confluence. The cells were washed three times with PBS, and a mixture of 1% Lucifer yellow (L0144; Sigma-Aldrich, MO, USA) was applied to the plates. Scratches were made on the plates by scoring to facilitate dye entry. Following a brief incubation, cells were washed with PBS and fixed with 3.5% paraformaldehyde for 10 minutes. The plates were sealed and visualized using FV10i confocal microscope (Olympus, Tokyo, Japan). And then the distance between the designated cut and the dye transfer was measured. This distance reflects cell activity, indicating that the dye passed to the neighboring cells.

### Protein half-life assay

As previously described 32, The cells that were transfected with the control siRNA or QRICH1 siRNA for 48 h were subjected to treatment with 50 μg/ml of Cycloheximide (CHX) (Merck, Darmstadt, Germany), an inhibitor of protein synthesis, for the indicated times prior to being collected and protein stability was assessed by Western blotting.

### Co-Immunoprecipitation (Co-IP)

As previously described 33, for co-immunoprecipitation (co-IP), 300 μg whole cell lysates from indicated cells were incubated with antibodies against QRICH1, USP1, Snail1, or Flag-tag at 4 °C overnight. The following day, 15 μl protein A/G agarose beads (sc-2003, Santa Cruz, TX, USA) were added to the antibody-lysate mixtures and incubated for an additional 2 h at 4 °C with gentle rotation. The resulting immunoprecipitates were washed three times with lysis buffer and analyze by SDS-PAGE and immunoblotting.

### Ubiquitination assay

As previously described 34, HEK293 cells were cotransfected with various HA-ubiquitin and the indicated gene overexpression plasmids. 48 h after transfection, 20 μl of 20 μM MG132 (474787; Sigma-Aldrich, MO, USA) was added to the plates for 4 h prior to harvesting. Whole-cell lysates were subjected to IP assays with indicated antibodies and immunoblotted.

### Lentivirus infection and stable cell line establishment

As previously described 35, to achieve stable knockdown, HEK293 cells were seeded onto culture plates the day before transfection. Lentiviral particles expressing QRICH1 shRNA (5'-TTTGGAAGCAGAAGTTCTG-3') or control shRNA purchased from GipZ (horizon discovery, UK) were transfected in HEK293 cells. Lentivirus supernatants collected from HEK293 cells were utilized to infect Huh7 cells, followed by selection in the presence of puromycin (100 μg/ml) for 2 weeks. Knockdown efficiency of QRICH1 was confirmed by qRT-PCR and western blotting.

### Animals

Five-week-old male athymic BALB/c nude mice supplied from JABIO (Suwon, Korea) were given to food and water *ad libitum*. They were housed four per cage with air-filtered lids under pathogen-limiting and controlled conditions at 22 ± 3˚C, with 50 ± 20% relative humidity and light set at 12 h/day (light on at 07:00). The condition of the mice was checked daily, and a humane endpoint was defined as a weight loss of more than 15% or a tumor volume of more than 1500 mm³. We also determined to stop the experiment if the mice developed severe ulceration or inflammation. All researchers have received special training on how to alleviate pain or fear in animals.

### Xenograft tumor model

Animal study was executed based on the protocol approved by Institutional Animal Care and Use Committee, Kyung Hee University (IACUC number: KHSASP-25-225). Huh7 cells were infected with control shRNA or QRICH1 shRNA. Once the cells were collected, 2 × 10⁶ cells suspended in 100 μl PBS were subcutaneously injected into the right flank of five-week- old male athymic BALB/c nude mice. The mice were monitored every day. After thirty days, the mice were sacrificed, and the tumors were excised and weighted. The tumor volume was determined as follows: volume = (length × width2) / 2. IHC was performed on the tumor tissues with antibodies of QRICH1, USP1, Snail1, PCNA, VEGF, E-cadherin and N-cadherin.

### Statistical analysis

The data in all graphs was analyzed by using GraphPad Prism software (Version 8.0.2, California, USA). All experiments were performed in triplicates and expressed as means ± standard deviation (SD). Statistical significance is determined using GraphPad Prism software, applying 1-way ANOVA with Dunnett's post-test for multiple comparisons. Student's t-test (unpaired, two-tailed) was used for two group comparisons. The P value of <0.05 is considered significant between groups.

## Results

### QRICH1 overexpression predicts poor prognosis in liver cancer patients, endogenous expression level in liver cancer cell lines and overexpression in liver cancer tissues

**Figure 1:**
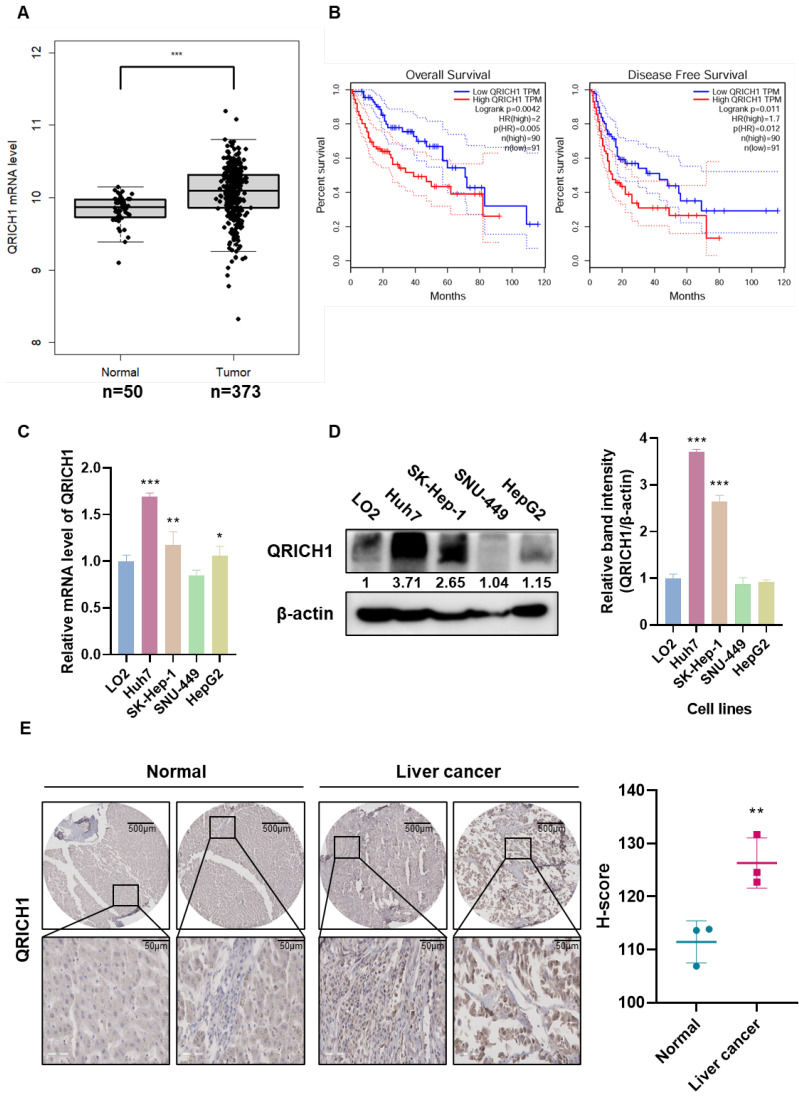
** QRICH1 overexpression correlates with poor prognosis and is elevated in liver cancer cell lines and tumor tissues.** (A) QRICH1 expression in normal tissue (n=50) and liver cancer (n=373) at mRNA levels. ***p<0.001 vs normal tissues. (B) Overall survival and Disease-free survival rates by Kaplan Meier plotter. (C) mRNA expression level in liver cancer cell lines. *p<0.05, **p<0.01, ***p<0.001 vs normal cells. (D) Protein expression level in liver cancer cell lines. ***p<0.001 vs normal cells. (E) Immunohistochemistry in normal and liver cancer tissues. Scale bar, 50 μm. **p<0.01 vs normal tissues.

*QRICH1* expression was significantly elevated in liver cancer patients according to TCGA-based bioinformatics analysis (Fig. 1A). Kaplan-Meier survival analysis further demonstrated that patients with high QRICH1 expression exhibited significantly poorer overall survival (OS) and Disease-Free Survival (DFS) (Fig. 1B), suggesting that QRICH1 upregulation is clinically relevant and potentially associated with a malignant tumor phenotype. Consistently, endogenous *QRICH1* levels were found to be substantially higher in the liver cancer cell lines Huh7 and SK-Hep-1 compared to normal hepatic cells, as confirmed at both the mRNA level (Fig. 1C) and protein level (Fig. 1D). These findings indicate that QRICH1 overexpression is not only observed in patient samples but is also maintained in hepatocellular carcinoma cell models. In addition, tissue array revealed stronger QRICH1 staining intensity compared to adjacent normal liver tissues (Fig. 1E), further validating that QRICH1 is consistently upregulated in tumor tissues. Together, these results strongly support the notion that QRICH1 is aberrantly overexpressed in liver cancer and may contribute to tumor progression.

### QRICH1 depletion reduced proliferation, migration in Huh7 and SK-Hep-1 cells

**Figure 2:**
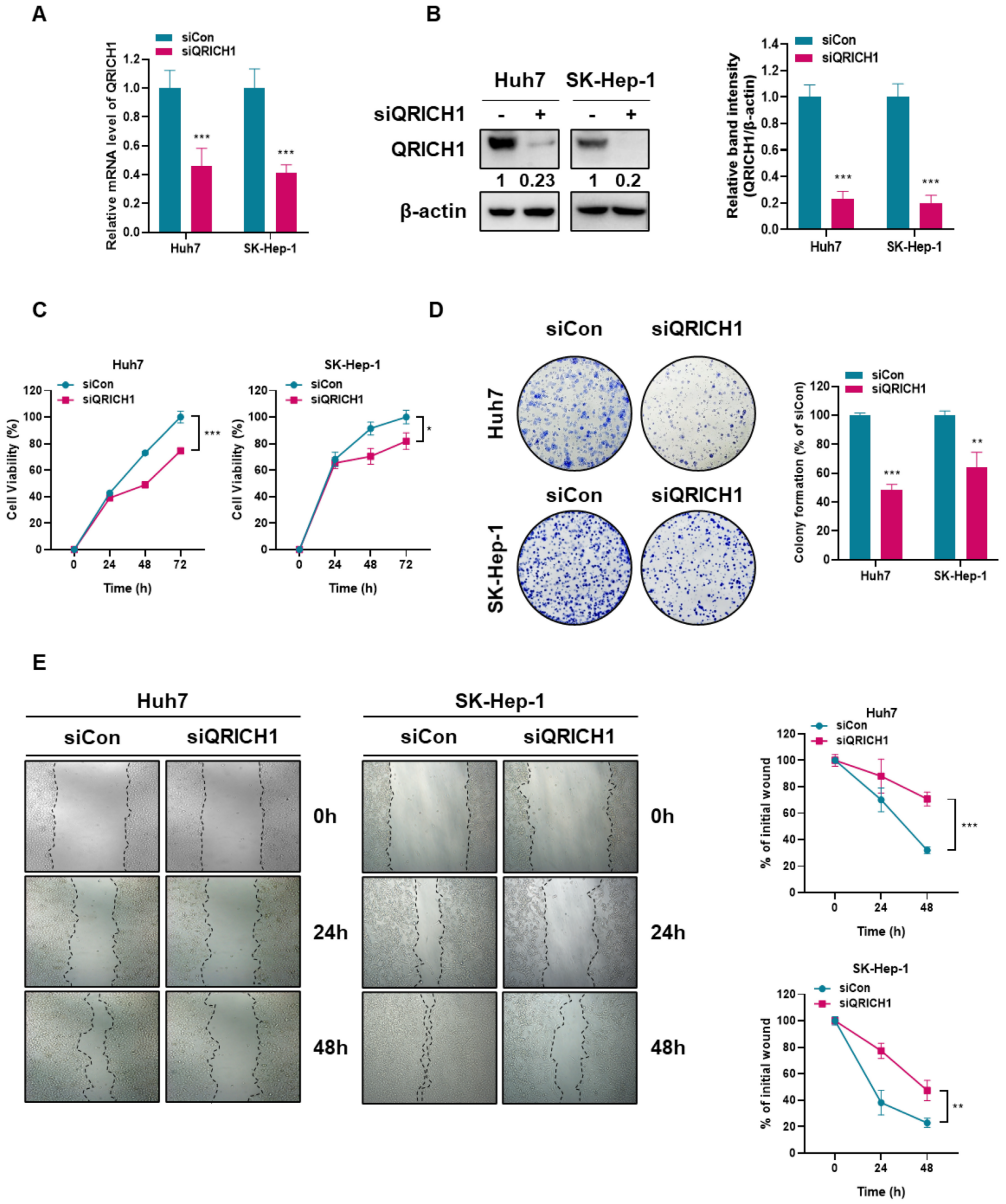
** Depletion of QRICH1 decreases proliferative and migratory abilities in Huh7 and SK-Hep-1 cells.** (A) QRICH1 expression was confirmed in QRICH1-depleted Huh7 and SK-Hep-1 cells at mRNA levels. ***p<0.001 vs untreated control. (B) QRICH1 expression was confirmed in QRICH1-depleted Huh7 and SK-Hep-1 cells at protein levels. ***P<0.001 vs untreated control. (C) Effect of QRICH1 depletion on the viability of Huh7 and SK-Hep-1 cells. *p<0.05, ***p<0.001 vs untreated control. (D) Effect of QRICH1 depletion on the number of colonies in Huh7 and SK-Hep-1 cells. **p<0.01, ***p<0.001 vs untreated control. (E) Effect of QRICH1 depletion on the proliferation of Huh7 and SK-Hep-1 cells by wound healing assay. Scale bar, 200 μm. **p<0.01, ***p<0.001 vs untreated control.

To explore the effects of QRICH1 depletion on the proliferation and migration of Huh7 and SK-Hep-1 cells, colony formation and wound healing assays were conducted in Huh7 and SK-Hep-1 cells transfected with siCon or QRICH1 siRNA plasmid. QRICH1 depletion was confirmed in Huh7 and SK-Hep-1 cells at mRNA (Fig. 2A) and protein (Fig. 2B) levels. Although QRICH1 depletion weakly attenuated the viability of Huh7 and SK-Hep-1 cells compared with untreated control (Fig. 2C), it markedly reduced the colony-forming capacity of both cell lines (Fig. 2D), indicating an essential role for QRICH1 in sustaining long-term proliferative potential. Also, wound-healing assays demonstrated that QRICH1 depletion significantly impaired the migratory and proliferative abilities of Huh7 and SK-Hep-1 cells (Fig. 2E). These findings suggest that QRICH1 promotes the proliferation and migration of liver cancer cells.

### Differentially expressed gene profiling in QRICH1-depleted Huh7 cells and close association between QRICH1 and Snail1

**Figure 3:**
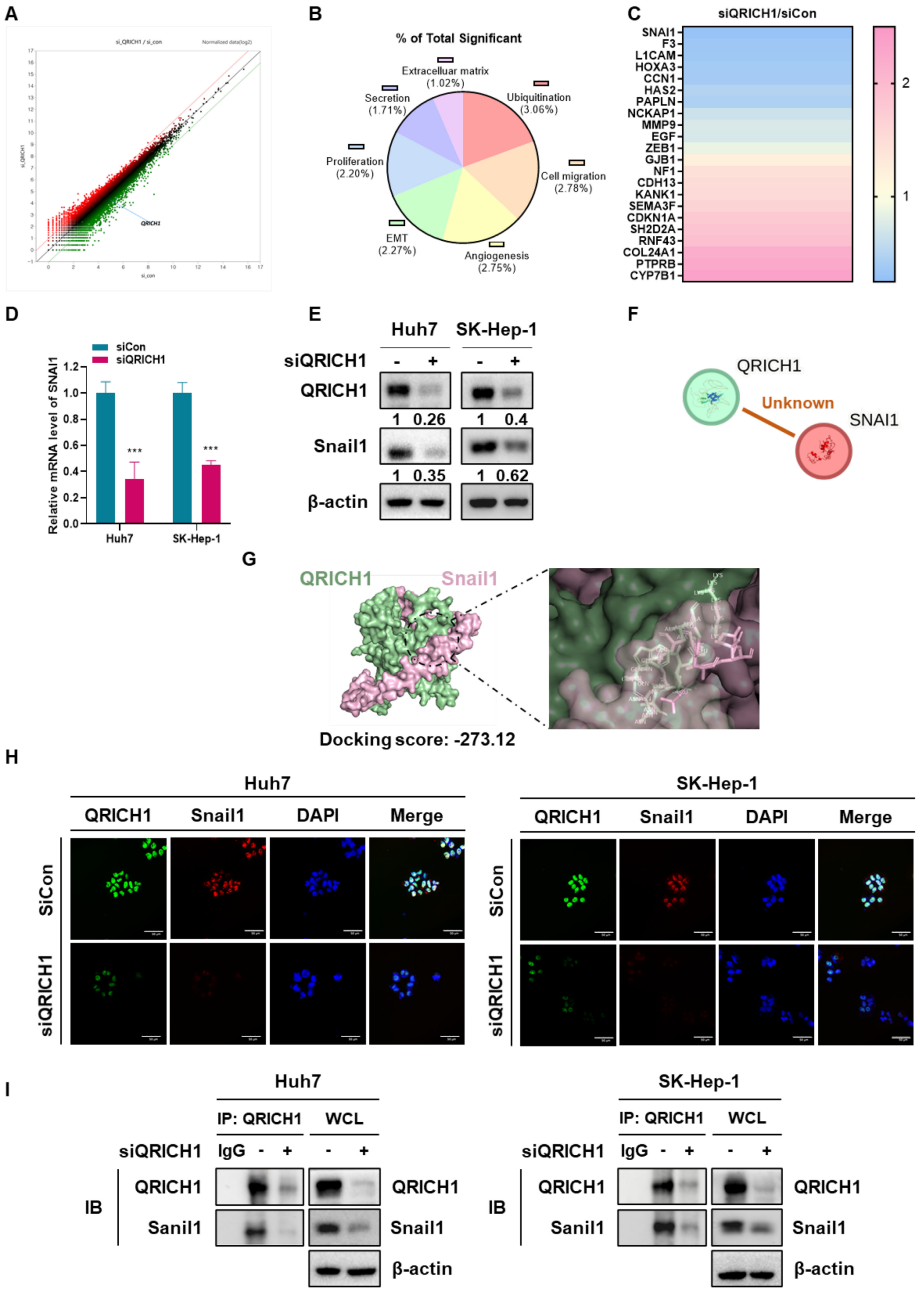
** Gene profiling of QRICH1-depleted Huh7 cells and close association between QRICH1 and Snail1.** (A) Heat map for differential gene expression in QRICH1-depleted Huh7 cells. (B) Percentages for signaling pathways in QRICH1-depleted Huh7 cells. (C) Differentially expressed genes in QRICH1-depleted Huh7 cells. (D) Effect of QRICH1 depletion on Snail1 in Huh7 and SK-Hep-1 cells by qRT-PCR analysis. ***p<0.001 vs untreated control. (E) Effect of QRICH1 depletion on Snail1 in Huh7 and SK-Hep-1 cells by Western blotting. (F) No evidence on the association between Snail1 and QRICH1 by String database. (G) Molecular docking study on a strong interaction between QRICH1 and Snail1. (H) Colocalization between Snail1 and QRICH1 by immunofluorescence. Scale bar, 50 μm. (I) Binding between Snail1 and QRICH1 by immunoprecipitation.

As shown in Fig. 3A, there were upregulated (pink) and downregulated (blue) gene profiles in QRICH1-depleted Huh7 cells. The signaling pathways were shown in ubiquitination (3.06%), cell migration (2.78%), angiogenesis (2.75%), EMT (2.27%) and proliferation (2.20%) (Fig. 3B). In order, downregulated genes are *SNAI1*, *F3*, *L1CAM*, *HOXA3*, *CCN1*, *HAS2*, *PAPLN*, *NCKAP1*, *MMP9*, *EGF* and *ZEB1*, while upregulated genes are *CYP7B1*, *PTPRB*, *COL24A1*, *RNF43*, *SH2D2A*, *CDKN1A*, *SEMA3F*, *CDH13*, *KANK1*, *NF1* and *GJB1* (Fig. 3C, Table 1). As shown in NGS data, QRICH1 depletion reduced the expression of *SNAI1* at mRNA (Fig. 3D) and protein (Fig. 3E) levels in Huh7 and SK-Hep-1 cells, though there was no evidence between QRICH1 and Snail1 by String database (Fig. 3F). Consistently, molecular docking analysis revealed a strong interaction between QRICH1 and the transcription factor Snail1, with a docking score of -273.12 kcal/mol (Table 2), suggesting a high binding affinity (Fig. 3G). Furthermore, QRICH1 was colocalized with Snail1 (Fig. 3H), and immunoprecipitation assays confirmed that QRICH1 directly binds to Snail1 in Huh7 and SK-Hep-1 cells (Fig. 3I). Collectively, the data support QRICH1 as a direct regulator of Snail1 expression.

### QRICH1 induces Snail1 protein stability in Huh7 and SK-Hep-1 cells

**Figure 4:**
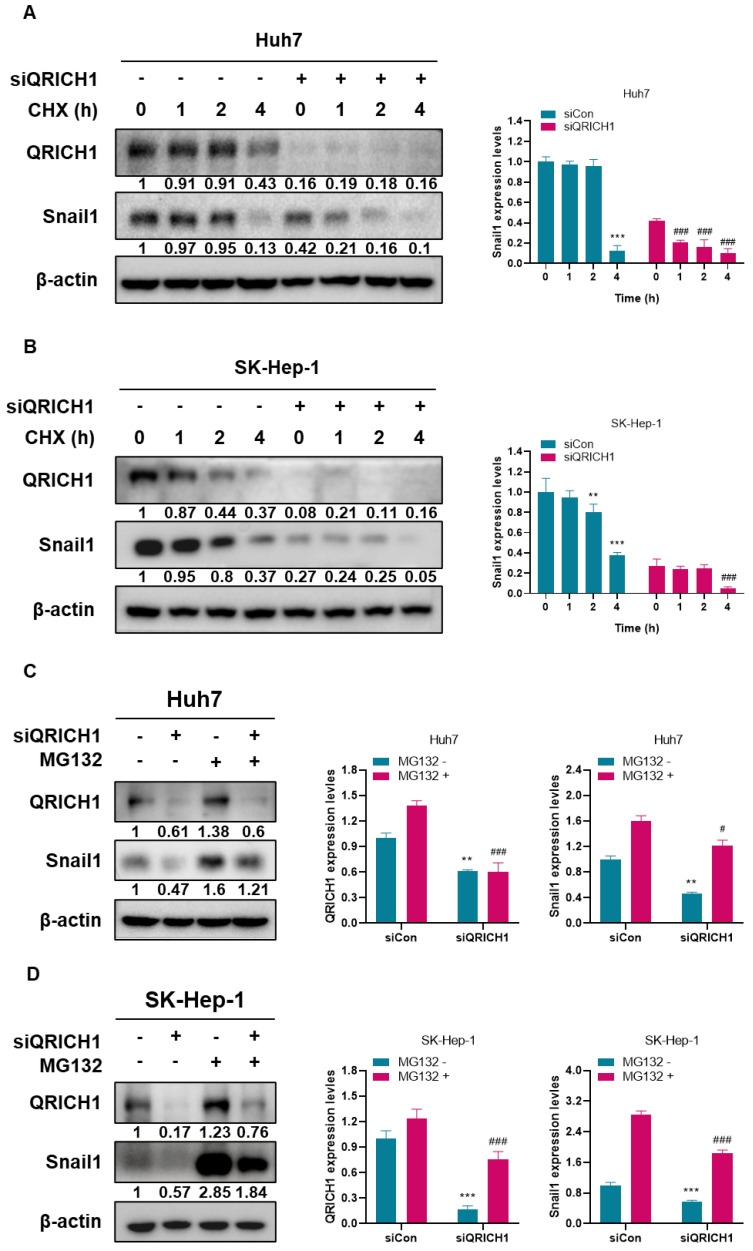
** Stabilization of Snail1 by QRICH1 in Huh7 and SK-Hep-1 cells.** (A) Effect of QRICH1 depletion on Snail1 expression in Huh7 cells. ***p<0.001 vs untreated control at 0 h, and ###p<0.001 vs Huh7 cells transfected with siQRICH1 at 0 h. (B) Effect of QRICH1 depletion on Snail1 expression in SK-Hep-1 cells. **p<0.01, ***p<0.001 vs untreated control at 0 h, and ###p<0.001 vs Huh7 cells transfected with siQRICH1 at 0 h. (C) MG132 rescues the inhibitory effect of QRICH1 depletion on Snail1 in Huh7 cells. **p<0.01 vs untreated control without MG132 and #p<0.05, ###p<0.001 vs untreated control with MG132. (D) MG132 rescues the inhibitory effect of QRICH1 depletion on Snail1 in SK-Hep-1 cells. ***p<0.001 vs untreated control without MG132 and ###p<0.001 vs untreated control with MG132.

To examine whether QRICH1 regulates Snail1 protein stability, cycloheximide (CHX) chase assay was performed in Huh7 and SK-Hep-1 cells. Following CHX treatment, Snail1 protein levels markedly declined in QRICH1-depleted Huh7 and SK-Hep-1 cells compared with control cells (Fig. 4A, B). In contrast, control cells maintained relatively stable Snail1 protein levels over the same time course, indicating that QRICH1 contributes to Snail1 protein maintenance. To determine whether this effect was mediated through proteasomal degradation, cells were treated with MG132, a selective proteasome inhibitor. MG132 treatment led to a pronounced accumulation of Snail1 protein; however, this effect was substantially attenuated in QRICH1-depleted Huh7 (Fig. 4C) and SK-Hep-1 cells (Fig. 4D). These findings suggest that QRICH1 promotes Snail1 protein stability by limiting proteasome-dependent degradation as a critical regulator of Snail1 turnover in liver cancer cells.

### Effect of QRICH1 depletion on EMT, cytoskeleton related proteins and GJIC in Huh7 and SK-Hep-1 cells

**Figure 5:**
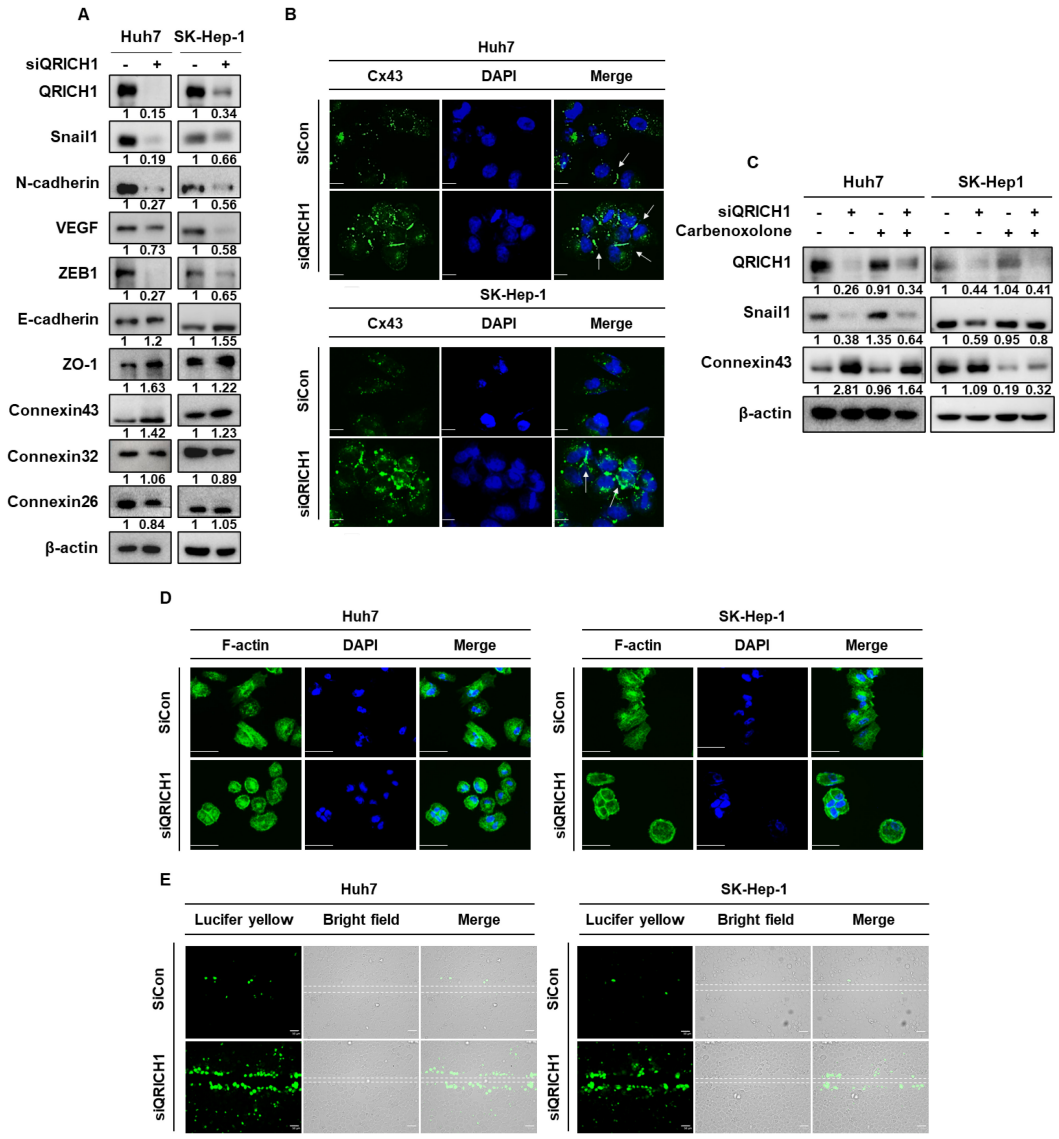
** QRICH1 depletion alters EMT, cytoskeleton dynamics, and GJIC in liver cancer cells.** (A) Effects of QRICH1 depletion on EMT-related and cytoskeleton-associated proteins, including Snail1, in Huh7 and SK-Hep-1 cells. (B) Effect of QRICH1 depletion on Connexin43 in Huh7 and SK-Hep-1 cells. Scale bar, 12.5 μm. (C) Effects of carbenoxolone treatment on QRICH1-regulated Snail1 and Connexin43 expression following QRICH1 depletion in Huh7 and SK-Hep-1 cells. (D) Effect of QRICH1 depletion on F-actin morphology in Huh7 and SK-Hep-1 cells by immunofluorescence. Scale bar, 50 μm. (E) Effect of QRICH1 depletion on GJIC green flow in in Huh7 and SK-Hep-1 cells by SL/DT assay. Scale bar, 50 μm.

To assess whether QRICH1 regulates EMT, the expression of key EMT- and cytoskeleton-related proteins was examined in Huh7 and SK-Hep-1 cells. QRICH1 depletion attenuated the expression of mesenchymal markers, including Snail1, N-cadherin, VEGF, ZEB1 and ZO-1, while upregulating epithelial markers and cytoskeleton related proteins such as E-cadherin and Connexin 43 (CX43) in Huh7 and SK-Hep-1 cells. In contrast, other connexin family members, including Connexin 32 (CX32) and Connexin 26 (CX26) were not affected by QRICH1 depletion (Fig. 5A). Interestingly, immunofluorescence analysis showed that QRICH1 depletion not only increased Connexin43 expression in both Huh7 and SK-Hep-1 cells but also enhanced its membrane localization and activation (green), thereby promoting gap junction intercellular communication (GJIC) compared with untreated controls (Fig. 5B). However, treatment with a GJIC blocker carbenoxolone reversed the effects of QRICH1 depletion, by suppressing the reduction of Snail1 and the activation of Connexin43 in Huh7 and SK-Hep-1 cells (Fig. 5C). Moreover, QRICH1-depleted Huh7 and SK-Hep-1 cells exhibited a pronounced shift from an elongated, mesenchymal-like phenotype to a more rounded, epithelial-like morphology (Fig. 5D). Consistently, QRICH1 depletion significantly increased in gap junctional intercellular communication in both Huh7 and SK-Hep-1 cells. This was demonstrated by the scrape loading and dye transfer (SL/DT) assay, in which QRICH1-depleted cells exhibited substantially greater green dye transfer compared with control cells (Fig. 5E).

### QRICH1 depletion downregulates USP1 and Snail1 expression in Huh7 and SK-Hep-1 cells through the disruption of its binding to USP1 or Snail1

**Figure 6:**
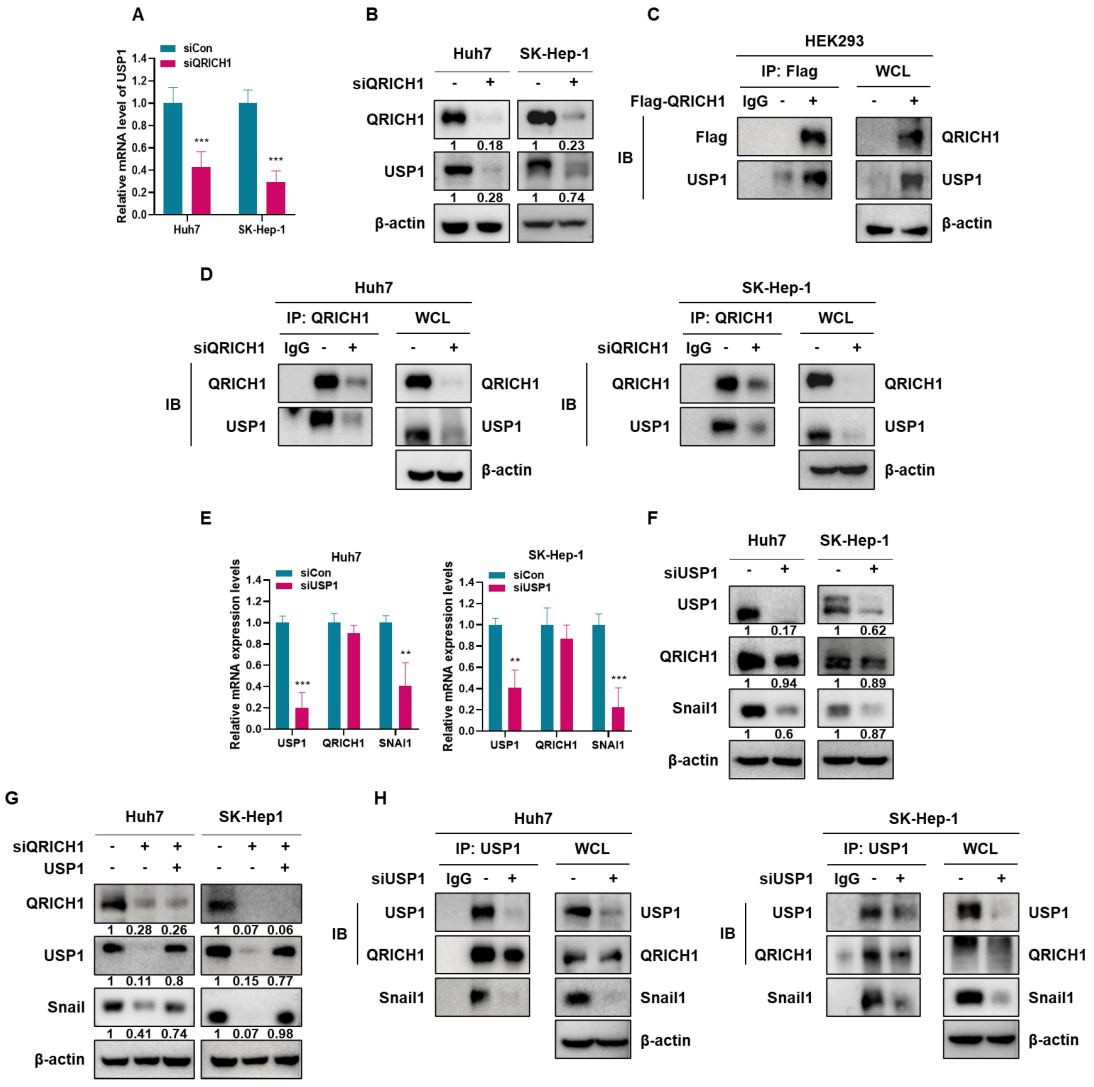
** Mechanistic association between QRICH1 and USP1 or Snail1 in Huh7 and SK-Hep-1 cells through their binding.** (A) Effect of QRICH1 depletion on USP1 expression in Huh7 and SK-Hep-1 cells by qRT-PCR. ***p<0.001 vs untreated control. (B) Effect of QRICH1 depletion on USP1 expression in Huh7 and SK-Hep-1 cells by Western blotting. (C) Interaction between QRICH1 and USP1 in HEK293 cells by Flag IP and IB. (D) The direct binding between QRICH1 and USP1 in Huh7 and SK-Hep-1 cells by IP. (E) Effect of USP1 depletion on QRICH1 and Snail1 expression in Huh7 and SK-Hep-1 cells by qRT-PCR. **p<0.01, ***p<0.001 vs untreated control. (F) Effect of USP1 depletion on QRICH1 expression in Huh7 and SK-Hep-1 cells by Western blotting. (G) Effect of QRICH1 depletion and USP1 overexpression on USP1 and Snail1 in Huh7 and SK-Hep-1 cells. (H) Effect of USP1 depletion on the binding to QRICH1 and Sanil1 in Huh7 and SK-Hep-1 cells.

It was well documented that USP1 has highest correlation coefficient (0.68) to regulate Snail1 among deubiquitination proteins (Table 3). Thus, to investigate the mechanistic association between QRICH1 and USP1 or Snail1, RT-PCR and immunoprecipitation (IP) analyses were performed in Huh7 and SK-Hep-1 cells. QRICH1 depletion significantly reduced the expression of *USP1* in Huh7 and SK-Hep-1 cells compared to untreated control at mRNA level (Fig. 6A) and protein level (Fig. 6B). Consistently, Flag immunoprecipitation followed by immunoblotting showed the interaction between QRICH1 and USP1 in HEK293 cells (Fig. 6C). Also, IP revealed that QRICH1 binds to USP1 in Huh7 and SK-Hep-1 cells compared to untreated control (Fig. 6D). In contrast, USP1 depletion weakly attenuated the expression of *QRICH1* but significantly suppressed the expression of *SNAI1* in Huh7 and SK-Hep-1 cells compared to untreated control at mRNA level (Fig. 6E) and protein level, despite significant disruption of Snail1 (Fig. 6F). QRICH1 depletion attenuated the expression of USP1 and Snail1, which was restored by USP1 overexpression in Huh7 and SK-Hep-1 cells (Fig. 6G), suggesting that QRICH1 regulates Snail1, at least in part, through USP1-mediated stabilization. Furthermore, it was confirmed that USP1 binds to QRICH1 or Snail1 in Huh7 and SK-Hep-1 cells compared to untreated control (Fig. 6H).

### QRICH1 depletion induced Snail1 degradation, which resulted from decreased USP1-mediated removal of K48-linked ubiquitin chains

**Figure 7:**
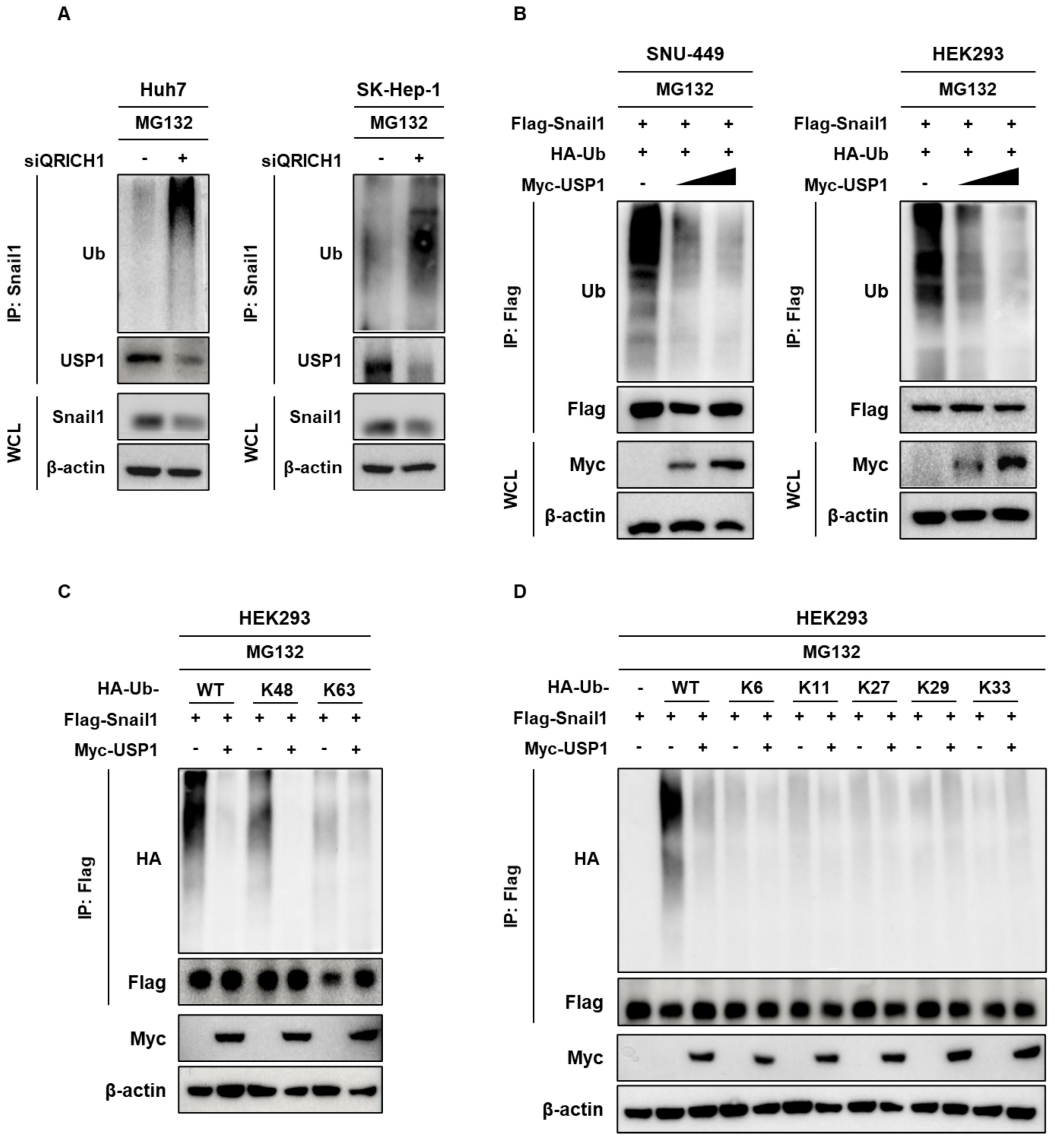
** USP1 dependent regulation of Snail1 ubiquitination and specification of ubiquitin chain linkages.** (A) Detection of Snail1 ubiquitination in Huh7 and SK-Hep-1 cells transfected with siQRICH1 and treated with MG132. (B) Assessment of ubiquitinated Snail1 in SNU-449 and HEK293 cells coexpressing the indicated vectors following a 4 h MG132 treatment, using Flag IP and subsequent IB analysis. (C) Determination of Snail1 ubiquitin-chain linkage patterns in HEK293 cells cotransfected with Flag-Snail1, Myc-USP1, and the designated His-Ub variants (WT, K48-only or K63-only). (D) Validation of ubiquitin-linkage in HEK293 cells expressing Flag-Snail1, Myc-USP1, and the indicated His-Ub plasmid (K6, K11, K27, K29, K33).

It is well known that MG132 as a proteasome inhibitor maintain Snail1 stability by blocking proteasomal degradation 36. Also, Snail protein is easily degraded through ubiquitination-mediated proteasome pathway 20. Here QRICH1 depletion induced ubiquitination in the presence of MG132 as a Snail1 stabilizer in in Huh7 and SK-Hep-1 cells (Fig. 7A). However, increasing concentrations of Myc-USP1 reduced the degradation of Snail1 in SNU-449 hepatocellular carcinoma cells and HEK 293 human kidney embryonic cells in the presence of MG132 and HA-Ub by IP (Fig. 7B). Ubiquitin is known to contain seven internal lysine residues (K6, K11, K27, K29, K33, K48, K63) and an N-terminal methionine (M1) 37. Interestingly, Snail1 degradation was induced in HEK293 cells at the sites of wild and K48, while USP1 as a deubiquitinating protein specifically removed K48-linked polyubiquitin chains, the canonical signal for proteasomal degradation to stabilizes its target Snail1 (Fig. 7C, 7D). Thus, given that USP1 removes K48-linked ubiquitin chains, QRICH1 depletion likely enhances the proteasomal degradation of Snail1.

### QRICH1 overexpression enhances proliferation and migration, accompanied by upregulation of EMT markers and cytoskeletal proteins in SNU-449 and HEK293 cells

**Figure 8:**
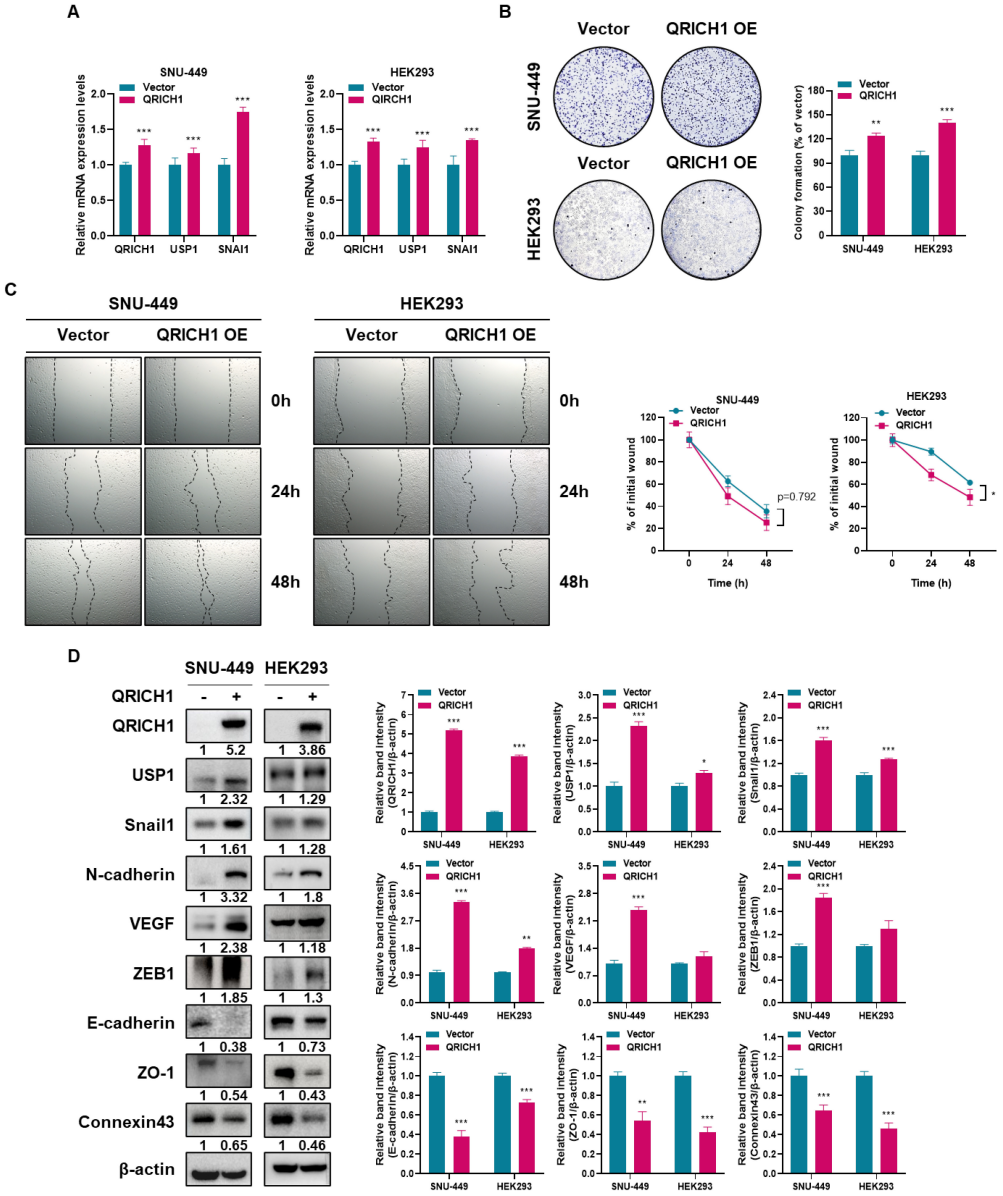
** Effect of QRICH1 overexpression on proliferation, migration and EMT and cytoskeleton related proteins in SNU-449 and HEK293 cells** (A) QRICH1, USP1, and Snail1 mRNA levels in SNU-449 and HEK293 cells transfected with QRICH1 overexpression plasmid. ***p < 0.001 vs. untreated control. (B) Clonogenic capacity of SNU-449 and HEK293 cells transfected with QRICH1 overexpression plasmid. **p<0.01, ***p<0.001 vs untreated control. (C) Migratory capacity of SNU-449 and HEK293 cells transfected with QRICH1 overexpression plasmid. Scale bar, 200 μm. *p<0.05 vs untreated control. (D) EMT- and cytoskeleton-related protein profiles of SNU-449 and HEK293 cells with QRICH1 overexpression. *p<0.05, **p<0.01, ***p<0.001 vs untreated control.

To determine the functional consequences of QRICH1 overexpression, SNU-449 which endogenously express QRICH1 at low levels, and HEK293 cells were transfected with a QRICH1 expression plasmid. SNU-449 and HEK293 cells transfected with QRICH1 overexpression vectors exhibited elevated mRNA levels of *QRICH1*, *USP1*, and *SNAI1*, consistent with previous findings (Fig. 8A). QRICH1 overexpression significantly increased the number of colonies in SNU-449 and HEK293 cells (Fig. 8B). Likewise, QRICH1 overexpression tends to increase the migration of SNU-449 and HEK293 cells, while it significantly enhanced the migration of SNU-449 and HEK293 cells by wound healing assay (Fig. 8C). Consistently, QRICH1 overexpression enhanced the expression of EMT-related proteins such as USP1, Snail1, N-cadherin, VEGF, ZEB1, and ZO-1, while reducing epithelial and cytoskeleton-associated proteins including E-cadherin and Connexin43 in SNU-449 and HEK293 cells (Fig. 8D).

### QRICH1 silencing attenuates the growth of Huh7 cells in Balb/c nude mice and regulates the expression of EMT and cytoskeleton related proteins

**Figure 9:**
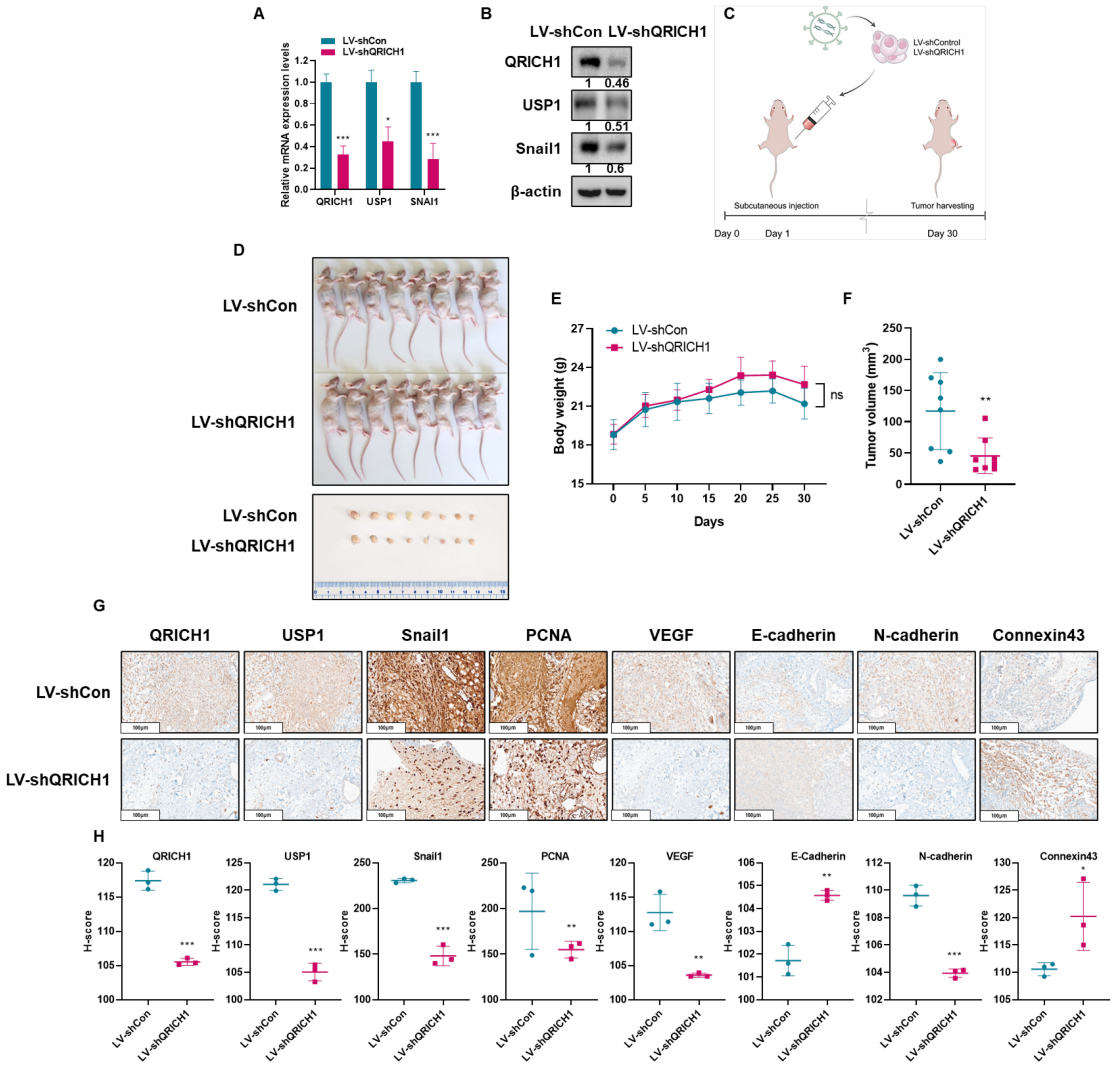
** QRICH1 depletion suppresses tumor growth and alters EMT and cytoskeletal protein expression in Balb/c nude mice bearing Huh7 xenografts.** (A) Quantification of QRICH1, USP1 and Snail1 mRNA levels in LV-shQRICH1-transduced Huh7 cells. *p<0.05, ***p<0.001 vs untreated control. (B) Protein expression of QRICH1 in QRICH1 depleted Huh7 cells. (C) Schematic timeline of the xenograft study in Balb/c nude mice. (D) Morphology of mice bearing Huh7 cells. (E) Body weight changes in mice implanted with QRICH1-depleted Huh7 cells. (F) Effect of QRICH1 depletion on tumor volumes of Huh7 cells in mice. *p<0.05 vs untreated control. **p<0.01 vs untreated control. (G) Immunohistochemical analysis of EMT- and cytoskeleton-related proteins in QRICH1-depleted Huh7 tumors. (H) H-score evaluation of IHC staining signals. *p<0.05, **p<0.01, ***p<0.001 vs untreated control.

To verify the *in vitro* observations at an *in vivo* setting, a xenograft model was established by implanting Huh7 cells transfected with LV-shQRICH1 lentiviral particles into Balb/c male athymic nude mice. Prior to implantation, the knockdown efficiency of the LV-shQRICH1 construct was verified at both the mRNA and protein levels (Fig. 9A, 9B). The experimental timeline for the xenograft procedure is presented in Fig. 9C. QRICH1 depletion significantly reduced the tumor volume of Huh7 cells compared to sham control (Fig. 9D, 9F) without hurting body weight. (Fig. 9E). IHC staining in LV-shQRICH1 tumors showed a pattern consistent with these observations. IHC evaluation revealed markedly reduced staining for QRICH1, USP1 (a deubiquitinating enzyme), PCNA (a proliferation and survival marker), and several EMT- or angiogenesis-related proteins, including Snail1, VEGF, and N-cadherin. In contrast, epithelial and junctional markers such as E-cadherin and Connexin43 showed enhanced immunoreactivity relative to the untreated control (Fig. 9G, 9H). Taken together, these *in vivo* data support an oncogenic role for QRICH1 and demonstrate that QRICH1 promotes tumor progression by modulating EMT and Connexin43-mediated junctional integrity.

## Discussion

In this study, we identified QRICH1 as a previously unrecognized driver of hepatocellular carcinoma (HCC) progression through its regulation of EMT and cytoskeleton related signaling pathways involving Snail1, USP1, and Connexin43. Although QRICH1 has been described as a stress-responsive protein associated with endoplasmic reticulum stress 14 and transcriptional regulation 15, its biological functions, particularly liver cancer, have remained largely unexplored.

Herein, QRICH1 was significantly overexpressed in liver cancer tissues with poor prognosis, suggesting its potential role as a clinically relevant oncogenic factor. Functional studies confirmed that QRICH1 depletion markedly reduced proliferation, colony formation, and xenograft tumor growth in Huh7 and SK-Hep-1 cells, underscoring its critical role for tumor maintenance and progression.

Next generation sequence revealed Snail1 was mostly downregulated in QRICH1-depleted Huh7 cells with the percentages of ubiquitination (3.06%), cell migration (2.78%), proliferation (2.20%), angiogenesis (2.75%), EMT (2.27%). Consistently, QRICH1 knockdown suppressed a broad range of EMT-related genes 38, 39, including SNAI1, F3, L1CAM, CCN1, PAPLN, MMP9, EGF, ZEB1 and activated cytoskeleton related genes such as CDH13 and GJB1^40^. The consistent downregulation of these key regulators suggests that QRICH1 acts as an upstream coordinator of EMT signaling networks. Notably, Snail1 emerged as a critical target of QRICH1, in line with its well-established role as one of the most potent EMT-inducing transcription factors in HCC 41, 42.

Mechanistically, our immunoprecipitation, immunofluorescence, and docking analyses revealed that QRICH1 directly interacts and colocalizes with Snail1. QRICH1 depletion increased the ubiquitination of Snail1 and promoted its proteasome-mediated degradation, which was restored by MG132. The accelerated decay of Snail1 in the presence of cycloheximide further confirms that QRICH1 stabilizes Snail1 at the post-translational level. Given the known role of USP1 as a deubiquitinase 43, our findings suggest that QRICH1 acts as an essential scaffold or enhancer that maintains the USP1/Snail1 regulatory axis, thereby sustaining EMT tumorigenic potential 44.

Gap junctional intercellular communication (GJIC) functions actively in most normal cells, whereas dysfunctional homologous or heterologous GJIC is frequently observed in cancer cells 45, 46. Also, Connexin 43, as a transmembrane protein or a gap junction protein 47 plays a dual role in acting either as a tumor suppressor 48 or in some contexts, as an oncogene 49, 50 through its regulation of intercellular communication. Furthermore, emerging evidence indicates that Cx43 at gap junctions interacts with drebrin for cancer cell invasion 51, stabilizing the protein at the plasma membrane and linking it to the actin cytoskeleton 24, 48.

Herein, QRICH1 depletion enhanced GJIC and induced a morphological shift from elongated mesenchymal-like cells to more oval epithelial-like shapes, accompanied by reduced Connexin43 expression. Although Connexin43 has context-dependent roles in cancer 11, its dysregulation is known to accompany EMT progression 12. Thus, the restoration of GJIC following QRICH1 knockdown represents another mechanism through which QRICH1 supports an oncogenic phenotype. Herein QRICH1 depletion upregulated Connexin43 as a tumor suppressor to enhance GJIC activity in Huh7 and SK-Hep-1 cells. In addition to the main signaling axis, depletion of Connexin43 resulted in increased Snail1 expression without affecting USP1 levels (Fig. S1), suggesting that Connexin43 suppresses EMT through regulation of Snail1 expression independently of USP1-mediated deubiquitination.

Previous evidence reveals that QRICH1 is known to regulate PRMT1-mediated arginine methylation of cGAS and is implicated in hepatocellular carcinoma progression 52. However, in our study, cGAS depletion reduced Snail1 expression without affecting USP1 levels, suggesting that USP1-Snail1 regulation is independent of the cGAS pathway and may be more directly regulated by QRICH1 (Fig. S2).

Our animal study supported the *in vitro* findings, as QRICH1 silencing suppressed tumor growth and reduced the expression of Snail1, USP1, N-cadherin, VEGF, and PCNA, while increasing E-cadherin and Connexin43 levels. These results confirm that the QRICH1 plays a crucial role in supporting liver cancer progression via Connexin43/USP1 mediated Snail1 stabilization axis.

Despite the strong mechanistic evidence, upstream regulators of QRICH1 and its role in other EMT signaling such as TGF-β, Wnt/β-catenin 53, or Notch signaling 54 should be further explored. Additionally, larger patient cohorts and clinical datasets are needed to validate QRICH1 as a prognostic biomarker and therapeutic target in liver cancer in the future.

## Conclusions

Collectively, our findings indicate that QRICH1 as a previously underappreciated regulator of liver cancer progression. Mechanistically, QRICH1 enhances Snail1 protein stability through a USP1-dependent deubiquitination pathway while concurrently influencing Connexin43-mediated intercellular communication. As a result of these interrelated processes, QRICH1 contributes to the activation of EMT programs that support malignant phenotypes in hepatocellular carcinoma. Importantly, this study establishes a functional link between EMT transcriptional regulation and gap junction signaling in liver cancer, providing mechanistic insight into how QRICH1 integrates these processes. Together, our results suggest that targeting QRICH1-centered signaling may represent a rational strategy for therapeutic intervention in HCC.

## Supplementary Material

Supplementary figures.

## Figures and Tables

**Table 1:** Differentially expressed genes in QRICH1-depleted Huh7 cells.

Gene Symbol	Function	Upregulation (Fold change)	Gene Symbol	Function	Downregulation (Fold change)
CYP7B1	metabolism/conditional	2.350	SNAI1	EMT/oncogene	0.295
PTPRB	Angiogenesis/suppressor	2.241	F3	Invasion/oncogene	0.343
COL24A1	Collagen/conditional	2.191	L1CAM	Adhesion/oncogene	0.347
RNF43	Proliferation/suppressor	1.920	HOXA3	EMT/oncogene	0.383
SH2D2A	*Immune/*conditional	1.862	CCN1	Proliferation/oncogene	0.388
CDKN1A	Cell-cycle/suppressor	1.784	HAS2	Invasion/oncogene	0.480
SEMA3F	Migration/Suppressor	1.628	PAPLN	ECM/oncogene	0.449
CDH13	Adhesion/suppressor	1.530	NCKAP1	Migration/oncogene	0.645
KANK1	Cytoskeleton/suppressor	1.504	MMP9	ECM/oncogene	0.742
NF1	Cell growth/suppressor	1.443	EGF	Growth-factor/oncogene	0.720
GJB1	Gap junction/ suppressor	1.208	ZEB1	EMT /oncogene	0.858

**Table 2:** Interaction between QRICH1 and Snail1 by molecular docking analysis.

Complex	Docking Score (kcal/mol)	Confidence Score	ΔG (kcal/mol^-1^)	Kd (M) at 37 ºC	Residue (QRICH1)	Residue (Snail1)	Distance (Å)	Interaction Type
QRICH1-Snail1	-273.12	0.9215	-16.5	2.2x10^-12^	LYS495	SER175	0.722	Polar
					ARG533	SER221	0.993	Polar
					GLU583	ARG224	1.905	Polar
					VAL575	LYS235	1.968	Polar
					TRP580	ARG224	2.039	Polar
					ARG576	TYR286	2.179	Polar
					ASN484	ALA168	2.313	Polar
					GLU579	GLN228	2.346	Polar
					ARG507	GLU256	2.512	Polar
					ALA491	HIS172	2.515	Polar
					GLU534	ARG191	2.556	Polar
					GLU492	SER190	2.61	Polar
					GLU494	LYS191	2.616	Polar
					ARG16	GLY261	2.631	Polar
					GLN487	TYR163	2.715	Polar
					ARG20	GLN255	2.767	Polar
					GLU75	GLN239	2.837	Polar

**Table 3:** Deubiquitinating proteins regulating snail1 stability.

Gene	Correlation coefficient	P-value
USP1	0.68	0
USP37	0.61	0
USP3	0.58	0
USP11	0.53	0
PSMD14	0.48	0
OTUB1	0.47	0
E1F3H	0.32	1.6e-10
USP18	0.3	5.9-09
USP27X	0.2	8e-05
USP17L2	0.18	0.00059
COPS5	0.17	0.0011
USP47	0.11	0.031
USP26	0.052	0.32

## Data Availability

The RNA-seq data generated in this study have been deposited in the Gene Expression Omnibus (GEO) under accession number GSE315229. All other data supporting the findings of this study are available from the corresponding author upon reasonable request.

## Abbreviations

cGAS: Cyclic GMP-AMP synthase
CHX: Cycloheximide
Co-IP: Co-immunoprecipitation assay
CX43: Connexin43
DUB: Deubiquitinating enzyme
ECL: Enhanced chemiluminescence
FBS: Fetal Bovine Serum
HCC: Hepatocellular carcinoma
IF: Immunofluorescence
IHC: Immunohistochemistry
MTT: 3-(4,5-dimethylthiazol-2-yl)-2,5-diphenyltetrazoliumbromide
NGS: Next-generation sequencing
OD: Optical density
PBS: Phosphate Buffered Saline
PCNA: Proliferating cell nuclear antigen
PRMT1: Protein arginine N-methyltransferase 1
QRICH1: Glutamine-rich protein 1
SDS: Sodium dodecyl-sulfate
SDS-PAGE: Sodium Dodecyl Sulfate-Polyacrylamide gel electrophoresis
SL-DT assay: Scrape Loading/Dye Transfer Assay
SNAI1: Snail Family Transcriptional Repressor 1
USP1: Ubiquitin Specific Protease 1
VEGF: Vascular endothelial growth factor
ZEB1: Zinc finger E-box binding homeobox 1
ZO-1: Zonula Occludens-1
