# Supplementary material for: Targeting QRICH1 suppresses epithelial-mesenchymal transition and tumor growth in liver cancer through the Connexin43/USP1 mediated Snail1 stabilization
Source: Int J Biol Sci. 2026 Jun 10;22(11):6177–95. doi: 10.7150/ijbs.133080 (PMC13282892; doi:10.7150/ijbs.133080)
Supplement: Supplementary file 1 — Supplementary figures. [file ijbsv22p6177s1.pdf]

Supplementary information for the manuscript

Targeting QRICH1 suppresses epithelial–mesenchymal transition and tumor growth in liver cancer through the Connexin43/USP1 mediated Snail1 stabilization

Su-Yeon Park, Bum-Sang Shim, Bonglee Kim, Sung-Hoon Kim

* Corresponding author: Sung-Hoon Kim, Cancer Molecular Targeted Herbal Research Laboratory, College of Korean Medicine, Kyung Hee University, 1 Hoegi-dong, Dongdaemun-gu, Seoul 02447, South Korea.

E-mail: sungkim7@khu.ac.kr; Tel: 82-2-961-9233; Fax: 82-2-961-9598

The file includes:

- Supplementary figures and figure legends

Supplementary figures

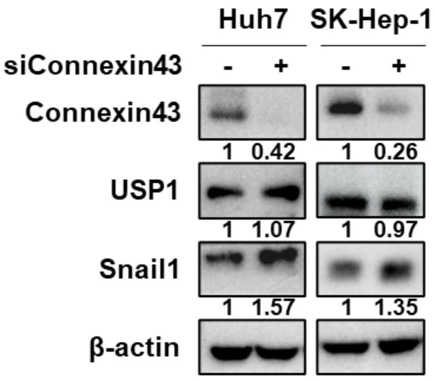

Figure S1. Connexin43 depletion upregulates Snail1 but not USP1 in Huh7 and SK-Hep-1 cells, indicating that Connexin43 suppresses Snail1 as an EMT regulator but does not directly regulate USP1, implying that USP1-mediated stabilization of Snail1 occurs independently of Connexin43.

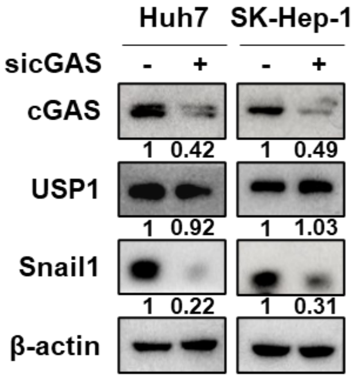

Figure S2. cGAS depletion downregulates Snail1 but not USP1 in Huh7 and SK-Hep-1 cells, indicating that cGAS positively regulates Snail1 expression without affecting USP1, suggesting that cGAS promotes EMT independently of USP1-mediated Snail1 stabilization.
